# Leptospirosis Complicated by Haemorrhagic Cardiac Tamponade: A Challenging Case of Weil’s Disease

**DOI:** 10.7759/cureus.99035

**Published:** 2025-12-12

**Authors:** Stephanie Walsh, Pamela McGibbon, Christopher Loew

**Affiliations:** 1 Anaesthetics/Critical Care, University Hospitals Dorset, Poole, GBR; 2 Critical Care, University Hospitals Dorset, Poole, GBR

**Keywords:** cardiac tamponade, case reports, haemorrhagic, leptospirosis, weil's disease

## Abstract

Leptospirosis is a bacterial infection with a worldwide distribution. It is most commonly transmitted through exposure to water contaminated with the urine of infected rodents. It is a biphasic illness consisting of an initial acute febrile phase lasting up to seven days, followed by a second immune phase with symptoms of organ involvement and widespread vasculitis lasting up to 30 days. Cardiac involvement, including arrhythmias and myopericarditis, is a recognised complication of leptospirosis. However, we present a case of haemorrhagic cardiac tamponade causing cardiac arrest, occurring 10 days after admission. This direct association is rarely reported. We hope to raise awareness to enable prompt recognition of this rare but serious complication. In addition, we feel that echocardiography during the immune phase of severe disease has the potential to improve outcomes.

## Introduction

Leptospirosis is a zoonotic infection with a wide range of clinical presentations. The causative organism, Leptospira, is part of the spirochaete family (also implicated in syphilis and Lyme disease). Important animal reservoirs include rats and small rodents, with humans typically becoming infected through water contaminated with the urine of an infected animal. Leptospira disseminate via the bloodstream, causing a vasculitis. Endothelial damage is thought to be due to both direct infiltration by Leptospira and the presence of immune complexes later in the disease process. However, the exact pathogenic mechanism remains unclear [1].

Many infections are asymptomatic or follow a mild non-specific febrile illness. The most common symptoms are fever, conjunctival suffusion, myalgia and headache. However, the most severe forms are icteric, rapidly progressing as a multisystem illness causing renal failure in Weil's disease or respiratory haemorrhage in ‘severe pulmonary haemorrhage syndrome’ [2].

Globally, its incidence is increasing, predominantly in low-income countries of tropical regions. In England, the diagnosis is rare with only 57 laboratory-confirmed cases annually between 2020 and 2023 [3]. More than 50% of these cases are acquired abroad with risk factors including recreational sports such as canoeing, exposure to heavy rainfall/flooding or occupational exposure to animals [4].

Given that leptospirosis often presents as a non-specific febrile illness, treatment is started based on clinical suspicion. In addition, diagnostic serology is usually not positive until six to 10 days after the onset of symptoms [5]. Leptospira are known to be sensitive to several antibiotics. However, it is now recognised that severe disease is likely to be immunologically mediated [6].

We present a case of haemorrhagic cardiac tamponade occurring in a 52-year-old gentleman, 10 days after admission with Weil’s disease. Cardiac complications present a diagnostic challenge as they occur in the immune-mediated phase of illness when the patient can appear to be recovering well. To our knowledge, this direct association between haemorrhagic cardiac tamponade and leptospirosis has only been reported in the literature once before [7].

## Case presentation

A 52-year-old gentleman presented to the emergency department with a seven-day history of feeling generally unwell with associated jaundice and abdominal pain following an alcohol binge. On examination, he was profoundly jaundiced, tachypnoeic, hypotensive, pyrexial and confused with minimal urine output. With no past medical history of note, the preliminary diagnosis was felt to be a first presentation of acute alcoholic hepatitis.

A computerised tomography (CT) scan of the abdomen and pelvis was performed that showed no specific abnormality of the pancreas, gallbladder or liver. The stomach and proximal small bowel were noted to be dilated; however, surgical review deemed this likely to be due to ileus as opposed to obstruction. Full blood count revealed a normocytic anaemia, thrombocytopaenia, neutrophilia and lymphopenia. CRP and procalcitonin (PCT) were both raised with a normal clotting time. Renal function was significantly deranged (acute kidney injury (AKI) stage 3) with a creatinine level of 841 µmol/l and urea of 50.9 mmol/l. Bilirubin was elevated at 470 µmol/l, albumin low at 19 g/l and a modest rise in alanine aminotransferase (ALT) of 181 units/l (Table 1).

**Table 1:** Admission blood results MCV: mean corpuscular volume, PLT: platelet, INR: international normalized ratio, eGFR: estimated glomerular filtration rate, ALT: alanine aminotransferase, ALP: alkaline phosphatase.

Test	Result	Reference Range
Haemoglobin	94 g/l	130-170 g/l
MCV	91 fl	78-99 fl
WBC	12 x10^9^/l	4.0-11.9 x10^9^/l
Neutrophils	7.2 x10^9^/l	2.0-7.5 x10^9^/l
Lymphocytes	0.1 x10^9^/l	1.5-3.5 x10^9^/l
Platelets	35 x10^9^/l	150-400 x10^9^/l
CRP	222 mg/l	0-9 mg/L
PCT	31.2 ng/ml	< 0.5 ng/ml
INR	1.1	0.9-1.1
Sodium	116 mmol/l	132-146 mmol/l
Potassium	5.5 mmol/l	3.5-5.0 mmol/l
Urea	50.9 mmol/l	2.5-6.7 mmol/l
Creatinine	841 µmol/l	45-110 µmol/l
eGFR	6 mL/min/1.73 m^2^	> 90 mL/min/1.73 m^2^
Albumin	19 g/l	35-48 g/l
Bilirubin	470 µmol/l	3-17 µmol/l
ALT	181 units/l	0-35 units/l
ALP	79 units/l	30-100 units/l

Due to progressive multiorgan failure, he was admitted to the critical care unit for close monitoring, blood pressure support and continuous venovenous haemodiafiltration (CVVHDF). The case was discussed with the Gastroenterology team who felt that the diagnosis of acute alcoholic hepatitis was unlikely. Subsequently, antibiotics were switched to ceftriaxone to cover for leptospirosis. Leptospirosis serology and PCR were sent. Potential exposure could be explained through his work as a security guard working at various job sites; no other risk factors were identified.

Further investigations including paracetamol levels, viral and autoimmune liver screen, vasculitis screen, HIV and myeloma screen came back negative. Paired bilirubin showed > 50% to be conjugated, with normal alkaline phosphatase (ALP) and no evidence of biliary obstruction. In the subsequent days bilirubin plateaued and started to decrease, thrombocytopenia, renal function and urine output improved supported by CVVHDF, and clinically, the patient improved. Leptospirosis serology and PCR came back as positive confirming the diagnosis of Weil's disease.

**Figure 1:**
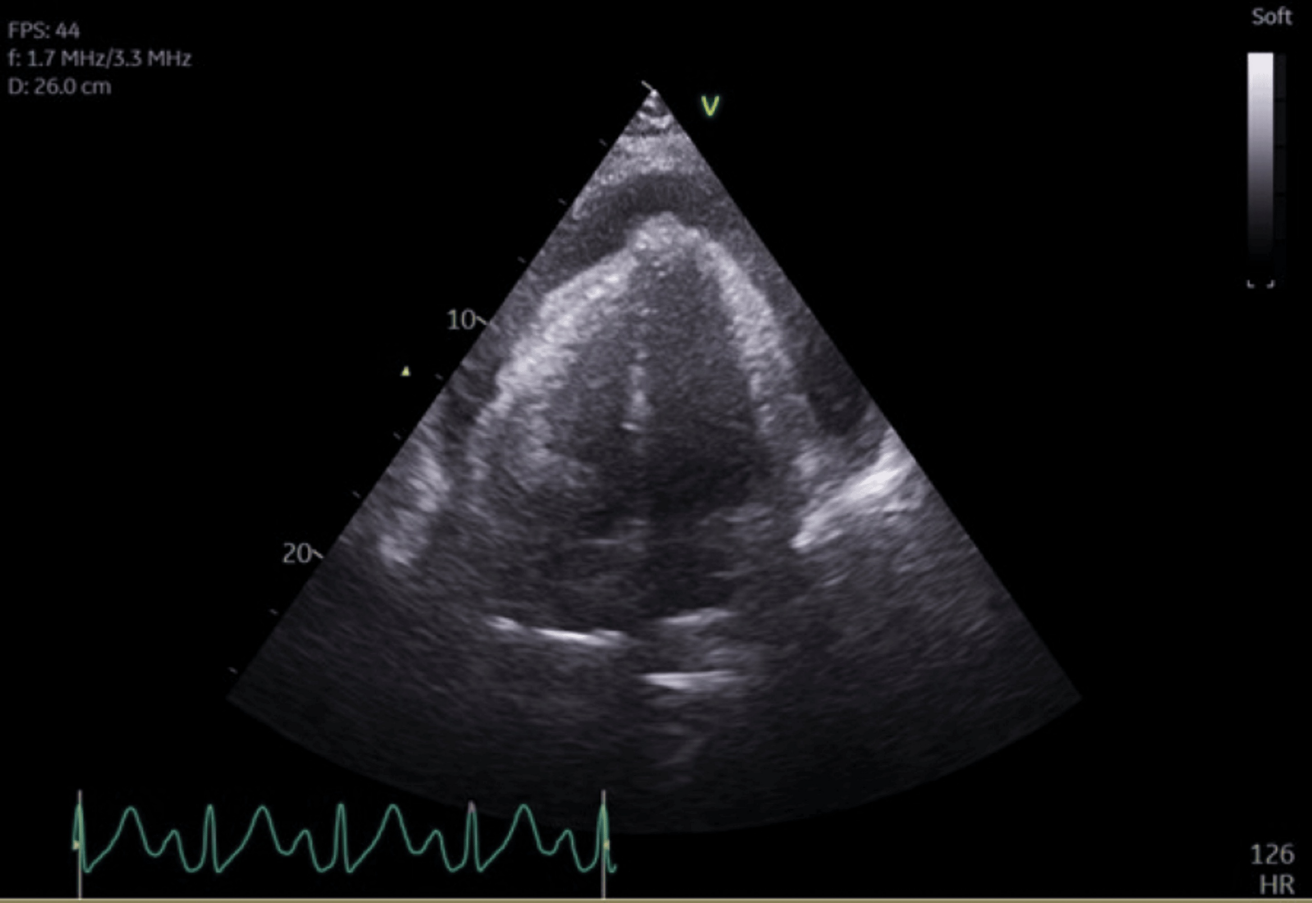
Apical four-chamber view showing a large global pericardial effusion FPS: frames per second, V: velocity, HR: heart rate, f: frequency, D: depth.

**Figure 2:**
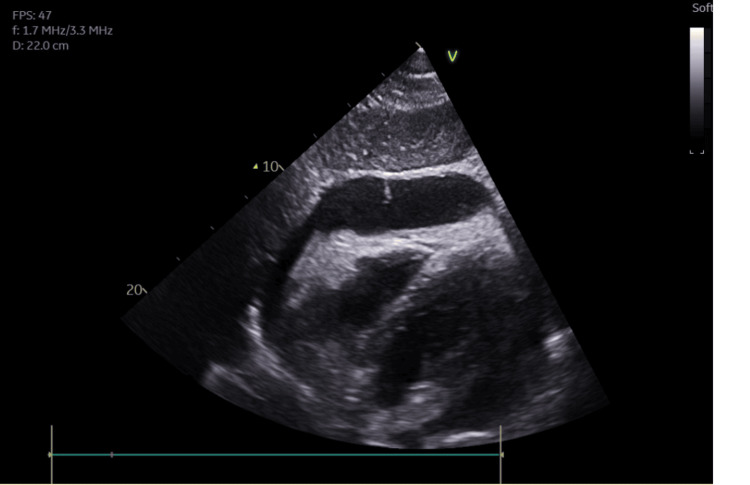
Subcostal view, 3.1 cm pericardial effusion around the right ventricle FPS: frames per second, V: velocity, f: frequency, D: depth.

Whilst still recovering on critical care, the patient had an acute episode of unresponsiveness. Blood gas revealed lactate of 9 with significantly reduced perfusion demonstrated by mottled extremities. ECG showed no evidence of ischaemia. Soon after, he became acutely unresponsive once again with a brief episode of tachycardia followed by witnessed asystole and cardiac arrest. Return of spontaneous circulation (ROSC) was achieved within eight minutes, and the patient was intubated and started on an adrenaline infusion. Bedside echocardiogram (ECHO) showed pericardial tamponade with right ventricle (RV) compromise (Figures 1, 2).

Pericardiocentesis was performed draining 250 ml of frank blood. Initially, the patient improved. However, several hours later, he became very unstable, hypotensive and adrenaline dependent with no pericardial drain output. Repeat ECHO showed re-accumulation of pericardial effusion with tamponade and presumed drain dislodgement. A further pericardial drain was inserted draining 600 ml immediately and then a further 700 ml of blood. Four units of RBC and four units of fresh-frozen plasma (FFP) were transfused empirically based on significant haemodynamic instability.

**Figure 3:**
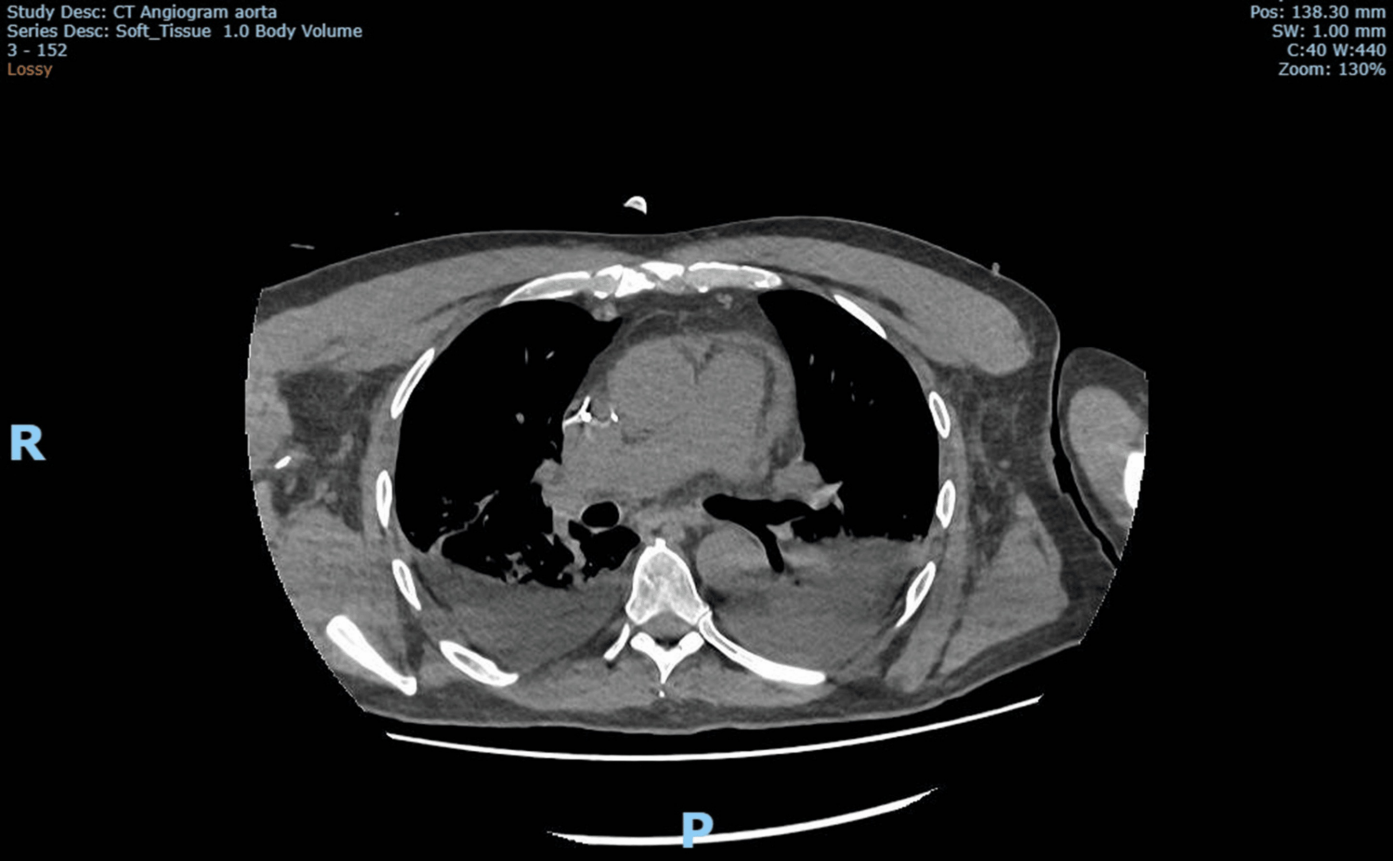
CT aortogram Pos: position, SW: section width, R: right, P: posterior.

Prior to cardiac arrest, coagulation parameters had been reported as international normalized ratio (INR) 1, activated partial thromboplastin time ratio (APTR) 1 and platelets 275 x10^9^/l. Citrate anticoagulation was used with CVVHDF, and the patient was on heparin 5,000 units twice a day (BD) as thromboprophylaxis. A CT aortogram was performed that showed evidence of the pericardial effusion and bilateral haemothorax but no evidence of thoracic aortic dissection (Figure 3).

After five days, the pericardial drain was removed, and the patient was extubated without complication. He continued to require dialysis under the renal team before being discharged from hospital.

## Discussion

Leptospirosis is a global public health concern which infects > 1 million people per year and is responsible for around 60,000 deaths [8]. The disease is found worldwide, with a much higher reported incidence in tropical regions, as the bacteria favour warm and humid conditions. Small mammals, specifically rats, are the most important reservoir hosts, whereby Leptospira bacteria are shed from the renal tubules into the urine. Humans may become infected through either direct contact with infected animals or indirectly through soil or water contaminated with infected urine [9]. An occupation that results in direct or indirect exposure is a significant risk factor for contracting the disease.

Clinical manifestations of leptospirosis infection range from a flu-like illness to life-threatening multiorgan failure. It is a biphasic illness whereby the first (anicteric) phase consists of high fever, headache, myalgia and conjunctival sufflation which coincides with leptospiraemia. After a brief afebrile period, the second (immune) phase ensues with resurgence of fever in addition to liver and kidney involvement. About 5%-10% of patients experience a severe form of the infection termed Weil's disease which is characterised by rapidly progressing renal and hepatic failure as with the case described here [10].

Cardiac manifestations of the disease are rarely reported in the literature [7,11,12]. However, an autopsy study in 2005 found that the cardiovascular system was involved in 93% of a total of 44 fatal cases. Examination of the hearts revealed cardiomegaly, petechiae on the epicardial surface and endocardial petechiae or haemorrhage. The predominant feature on histopathological examination was interstitial myocarditis. They concluded that in these cases, the cardiovascular involvement had been masked by the dominant pulmonary symptoms caused by pulmonary haemorrhage and/or diffuse alveolar damage [13]. In this case, there was no clotting abnormality detected at the time of cardiac arrest; therefore, the haemorrhagic complications are presumed to be as a result of the immune-mediated vasculitis associated with leptospirosis.

## Conclusions

We report this case with the hope of increasing awareness of haemorrhagic cardiac tamponade as a possible complication of leptospirosis. We also demonstrate the utility of skilled bedside ECHO in cardiac arrest. Despite being an uncommon diagnosis in the UK, this case highlights the potential for leptospirosis to cause multiorgan failure. It is an important consideration in the differential diagnosis of anyone presenting acutely unwell with jaundice. Early intervention in critical care with CVVHDF is vital in the supportive management of the illness. Cardiac complications of leptospirosis should be suspected in anyone with severe disease exhibiting haemodynamic instability in the immune phase of the illness.
